# An anatomical composite nasal lining subunit technique in primary cleft nose correction

**DOI:** 10.1016/j.jpra.2021.02.005

**Published:** 2021-03-06

**Authors:** Derek A. Mendonca

**Affiliations:** a Adjunct Clinical Associate Professor, Mohammed Bin Rashid University of Medicine and Health Sciences (MBRU), Dubai, United Arab Emirates; b Consultant Paediatric Plastic Surgeon, Department of Paediatric Plastic Surgery, Al Jalila Children's Hospital, PO Box 7662, Jaddaf, Dubai, United Arab Emirates

**Keywords:** Cleft nose, Primary, Unilateral, Cartilage lining subunit, Anatomical composite subunit

## Abstract

Current primary cleft nose correction techniques are associated with a significant rate of long term alar collapse. The nasal lining on the cleft side has been observed to be distorted and deficient. Nasal endoscopy was used to map the two dimensional topography of the anterior nasal airway lining in a normal and patient with unilateral cleft lip. The vestibular nasal subunit was noted to have a triple structural overlap (Lateral crus, valve and vestibule units). A nasal lining subunit based surgical strategy was designed, based on the subunit principle. The lateral crural tethering was released and differential repositioning of the cartilage/lining complex performed. The difference in domal height between the cleft and non-cleft sides was translated into a superior and medial advancement of the cartilage/lining composite subunit. The valve sub-unit defect was resurfaced with a vermilion full thickness graft, taken at the time of primary cleft lip repair. Primary septal relocation was performed and no percutaneous cartilage sutures were done. Pre and post-operative anthropometry measurements were obtained, and repeated at follow up. Complete nasal correction was seen in the unilateral cleft lip patient and was noted to be stable at 1 year follow-up. A novel nasal cartilage/lining subunit topographical map is proposed and forms the basis for a surgical strategy addressing comprehensive correction of the unilateral cleft nasal deformity.

## Introduction

Primary surgical correction of the cleft nasal deformity is associated with significant long term alar collapse^1^^.^ The cleft sided nasal cartilage is slumped and tethered laterally to the pyriform base.^1^^,^^2^ The author has observed that the nasal lining is abnormal on the cleft side, with clear differences between the normal versus unilateral cleft nose deformity. Starbuck et al.^3^ studied the anterior airway volume in patients with cleft nasal deformity, and objectively demonstrated that the anterior airway volume is reduced on the cleft side.

## Methods

**Figure 1:**
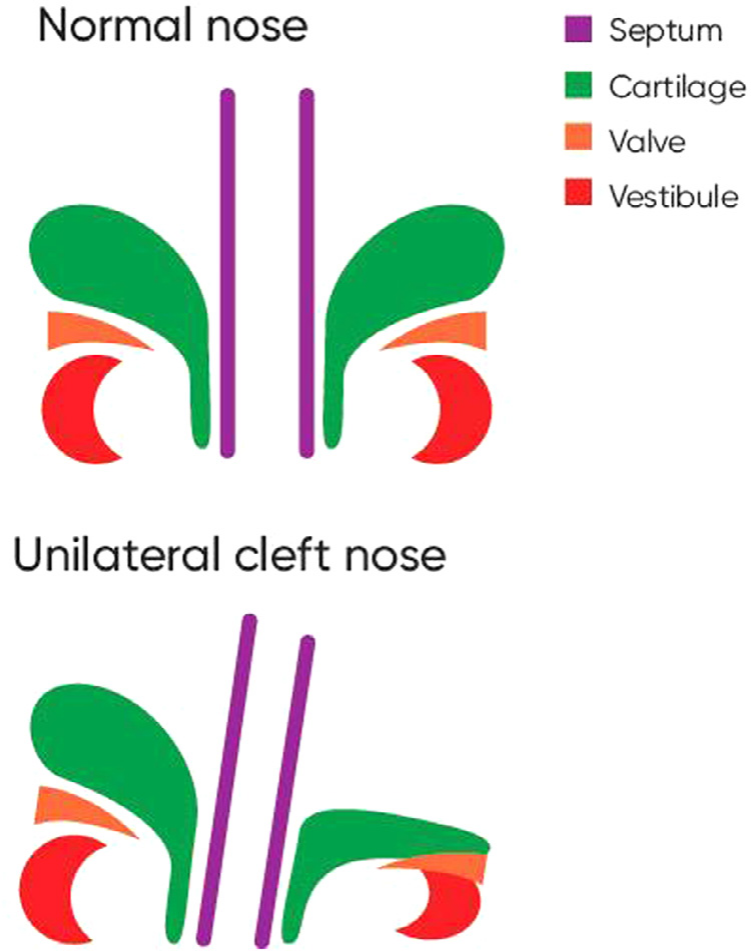
Topographical representation of nasal composite lining subunits in a normal versus a cleft nose.

The two dimensional topography of the anterior nasal airway lining was observed and mapped in a normal patient and patient with unilateral cleft lip. The cleft patient was incidentally undergoing diagnostic endoscopy for posterior airway pathology. An age matched patient with normal anterior nasal anatomy undergoing nasal endoscopy for other indications was used as a control. The vestibular nasal subunit was noted to be distorted and deficient in the patient with unilateral cleft lip (Figure 1). The lateral vestibule had a triple structural overlap (Lateral crus, valve and alar subunits). A subunit based surgical strategy was conceptualised based on the above findings.

### Surgical technique

Anthropometric landmarks^4^ are located on the cleft nose and lip. Subnasale (Sn), cleft and non-cleft nasal projection (prn), alar base (al) are marked in addition to the lip landmarks. Nasal correction is executed prior to lip surgery.

**Figure 2:**
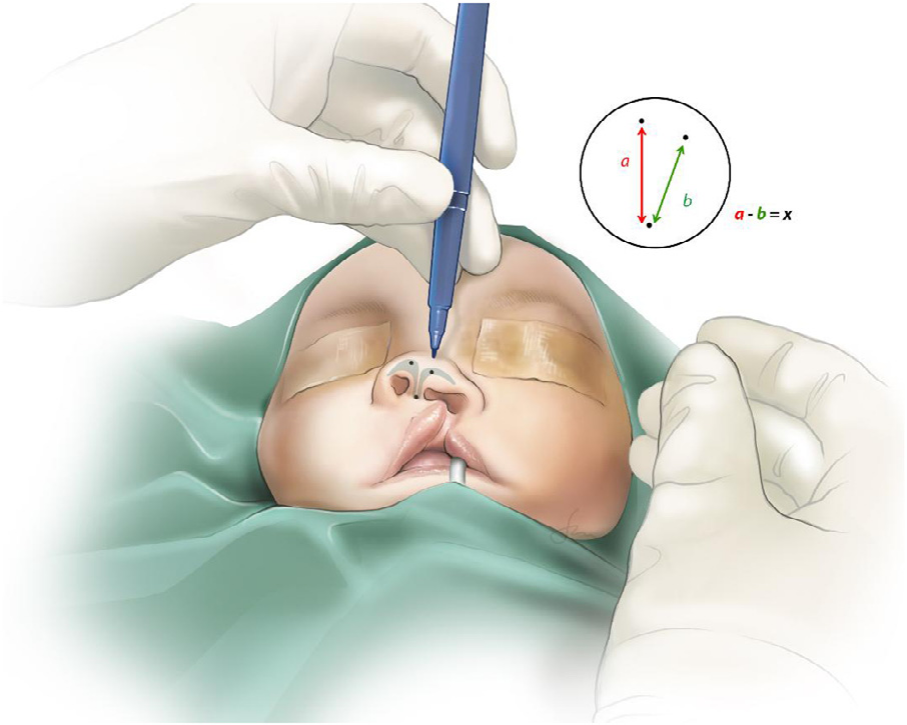
Marking of the cleft and non-cleft nasal heights as (a) and (b), with the difference as (x) in mm.

The heights of the non-cleft and cleft nasal projection (sn – prn) are measured as a and b (mm) respectively (Figure 2). The difference between a and b is x (mm).

The cephalic and caudal margins of the lower lateral cartilage are marked as two parallel lines within the vestibule. The insertion and tethering point of the cartilage is marked as a point at the lateral insertion, within the vestibule. The two parallel lines are incised and gentle dissection is done at the superior incision, in the plane above the cartilage, separating the attachments to the skin (Supplementary material). Limited nasal tip dissection is done to release the lateral crural attachments. The lateral tethering point is now incised releasing the composite unit. The complex is repositioned medially and superiorly by adding 4 mm to x. The distance of 4 mm is added to the medial movement based on study by Kim,^5^ that showed the cleft side medial crura is inferiorly placed by a mean distance of 4 mm. The composite lining complex is now repositioned in the new location, adjacent to the non-cleft dome and held in place with a 26 gauge needle. The composite lining complex is sutured to the new position with absorbable sutures (Supplementary material). A template of the vestibule defect is obtained to harvest the vermilion graft from the portion of the lip, which is traditionally discarded (M flap). The graft is thinned and inset meticulously into the vestibule defect. A key step is to insert a quilting suture through the graft, brought outside the nasal skin as a bolster suture. This step ensures constant graft wound bed contact, and increases the chances of graft survival. The next critical steps in the nasal repair are septal relocation and alar nasal muscle repair to the periosteum, recreating the alar curve (Supplementary material). An inter-domal suture is placed to reduce the tension across the domes, and a nostril stent is kept in place for 1–2 weeks.

## Results

**Figure 3:**
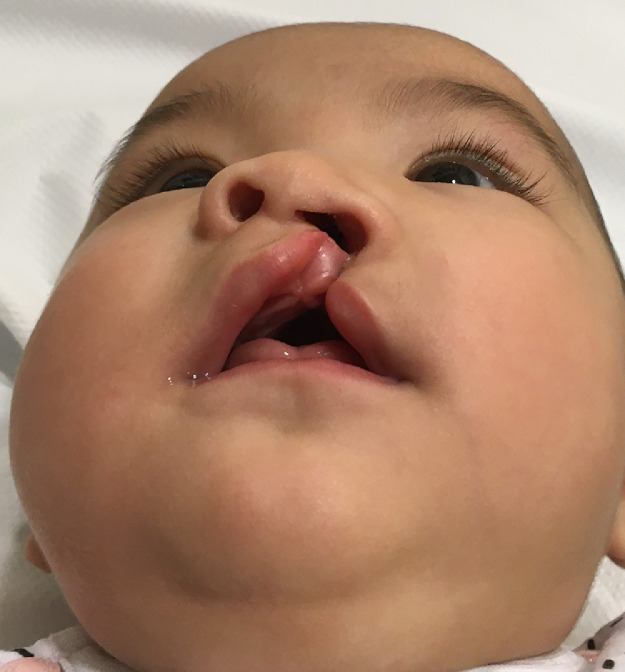
4 month old baby with cleft lip and nose deformity showing the nasal lining distortion.

**Figure 4:**
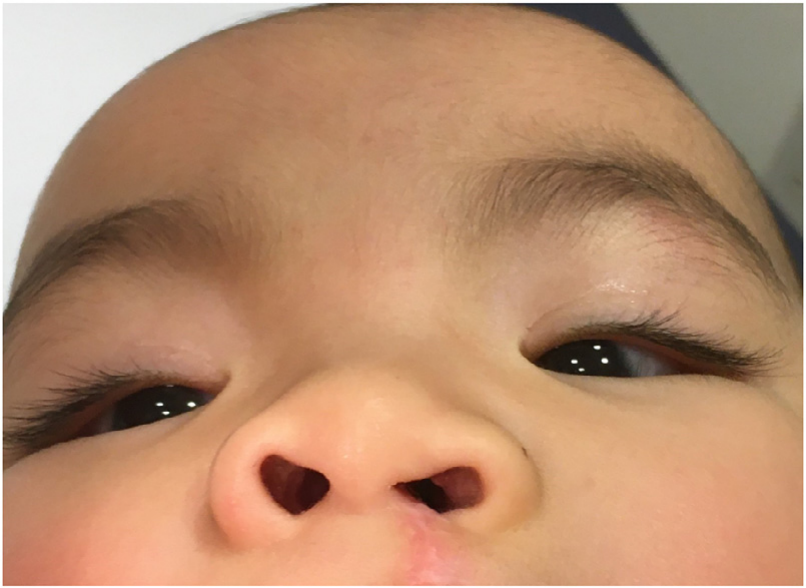
1 year follow up post anatomical nasal composite lining sub-unit repair.

The nasal lining sub-unit based corrective strategy was executed in a 4 month baby girl with unilateral cleft lip (Figure 3). Pre and post-operative anthropometry measurements (nasal width, nasal projection, and nostril width and nostril height) were obtained. Nasal projection, nasal height and width remained unchanged at follow up. Nasal correction added 32 minutes to the surgical duration of the lip repair. At 1 year follow up, there was no nasal stenosis, relapse or change in nasal correction (Figure 4).

## Discussion

Current primary nose correction techniques are associated with significant long term alar collapse and secondary rhinoplasty.^1^^,^^2^^,^^6^ Techniques such as extensive closed nasal dissection, percutaneous cartilage sutures, rim incisions and relocation of the deviated septum do not address the lateral tethering force.^2^^,^^3^^,^^7^ This deep adherent tissue combined with a lining deficiency is the key factor responsible for the nasal deformity.

Gillies and Millard^10^ described the foundational principles of plastic surgery: (1) release of abnormal tissues, (2) return and retention of abnormal tissues to its normal anatomical position, (3) borrowing from Peter to pay Paul and finally, (4) not discarding any useful tissues. These concepts have been incorporated in the design and philosophy of the current surgical technique.

Huang^7^ applied mathematical modelling to the forces of tension within the cleft nose. The study demonstrated maximum correction occurred with superior positioning of the dome. This correction can only happen with an internal surgical lining release and differential repositioning of the cartilage/mucosa complex. Techniques that address the lateral crural cartilage advance the composite lining unit, as local advancement flaps.^8^^,^^10^ These techniques rely on local flaps for closure from an area that is already deficient in lining tissue, therein resulting in a higher incidence of nasal stenosis and scar contracture. Rees^9^ described the use of a skin graft in the vestibule in secondary cleft nose deformities. The technique has been executed on 3 more patients, and further studies are ongoing.

The current technique differs from all the previous in the following:

1. One stage comprehensive primary cleft nose correction at the time of lip repair
2. Description of anatomical lining subunits with an anthropometric based plan for nasal correction
3. Differential supero-medial rotation of cartilage and mucosa opens the anterior nasal airway addressing the tissue deficiency in addition to, releasing the lateral tensioning force
4. Use of a vermilion full thickness graft to address vestibule tissue deficiency

## Conclusion

A novel nasal cartilage/lining subunit classification is proposed and forms the basis for a surgical strategy addressing comprehensive correction of the unilateral cleft nasal deformity.

## Declaration of Competing Interest

There is no conflict of interest with the publication of this manuscript. Full informed consent has been obtained by the patient's family for publication.

## Appendix Supplementary materials

Image, application 1

## References

[1] Millard D.R. (1976). Primary nasal correction. Cleft Craft.

[2] Wolfe S.A., Nathan N.R., MacArthur I.R. (2016 Jan). The cleft lip nose: primary and secondary treatment. Clin Plastic Surg.

[3] Starbuck J.M., Friel M.T., Ghoneima A., Flores R.L., Tholpady S., Kula K. (2014 Oct). Nasal airway and septal variation in unilateral and bilateral cleft lip and palate. Clin Anat.

[4] Farkas L.G. (1996 Jan). Accuracy of anthropometric measurements: past, present, and future. Cleft Palate Craniofac J.

[5] Kim Y.S., Cho H.W., Park B.Y., Jafarov M. (2008 Oct). A comparative study of the medial crura of alar cartilages in unilateral secondary cleft nasal deformity: the validity of medial crus elevation. Ann Plast Surg.

[6] Monson L.A., Kirschner R.E., Losee J.E. (2013 Dec). Primary repair of cleft lip and nasal deformity. Plast Reconstr Surg.

[7] Huang H., Luo X., Cheng X., Shi B., Li J. (2018 Jun 28). Biomechanical simulation of correcting primary unilateral cleft lip nasal deformity. PLoS ONE.

[8] Rossell-Perry P. (2017 May-Aug). Primary unilateral cleft lip nasal deformity repair using V-Y-Z plasty: an anthropometric study. Indian J Plast Surg.

[9] Rees T.D., Guy C.L., Converse J.M. (1966 Jan). Repair of the cleft lip-nose: addendum to the synchronous technique with full-thickness skin grafting of the nasal vestibule. Plast Reconstr Surg.

[10] Gillies H.D., Millard D.R. (1957).

