# Supplementary material for: An anatomical composite nasal lining subunit technique in primary cleft nose correction
Source: JPRAS Open. 2021 Mar 6;28:72–6. doi: 10.1016/j.jpra.2021.02.005 (PMC8027686; doi:10.1016/j.jpra.2021.02.005)
Supplement: Supplementary file 1 [file mmc1.docx]

Supplementary material 1: Topographical representation of nasal composite lining sub-units in a normal versus cleft nose


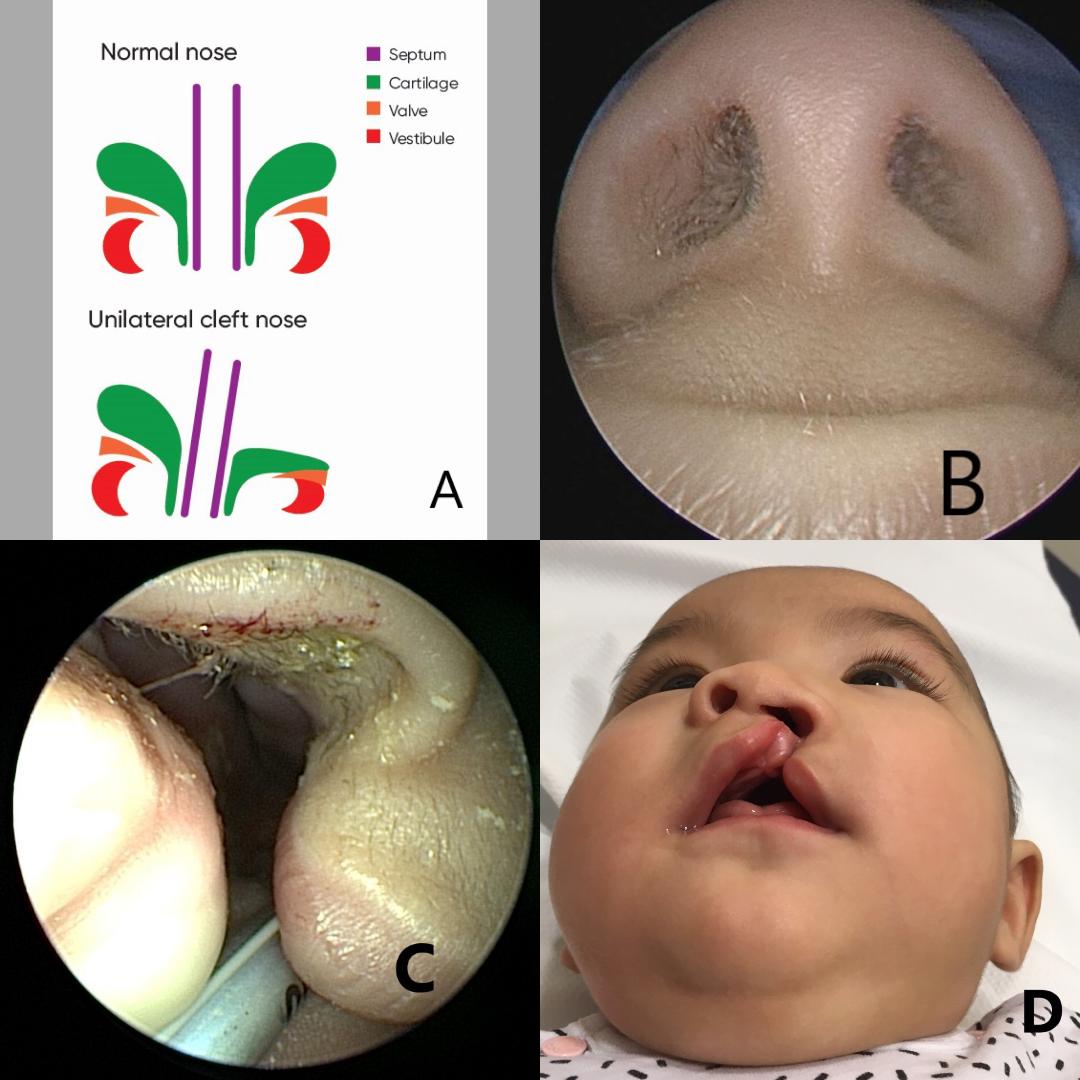


Supplementary material 2: Series of surgical steps showing the marking, incisions within the nose and release of composite lateral crus


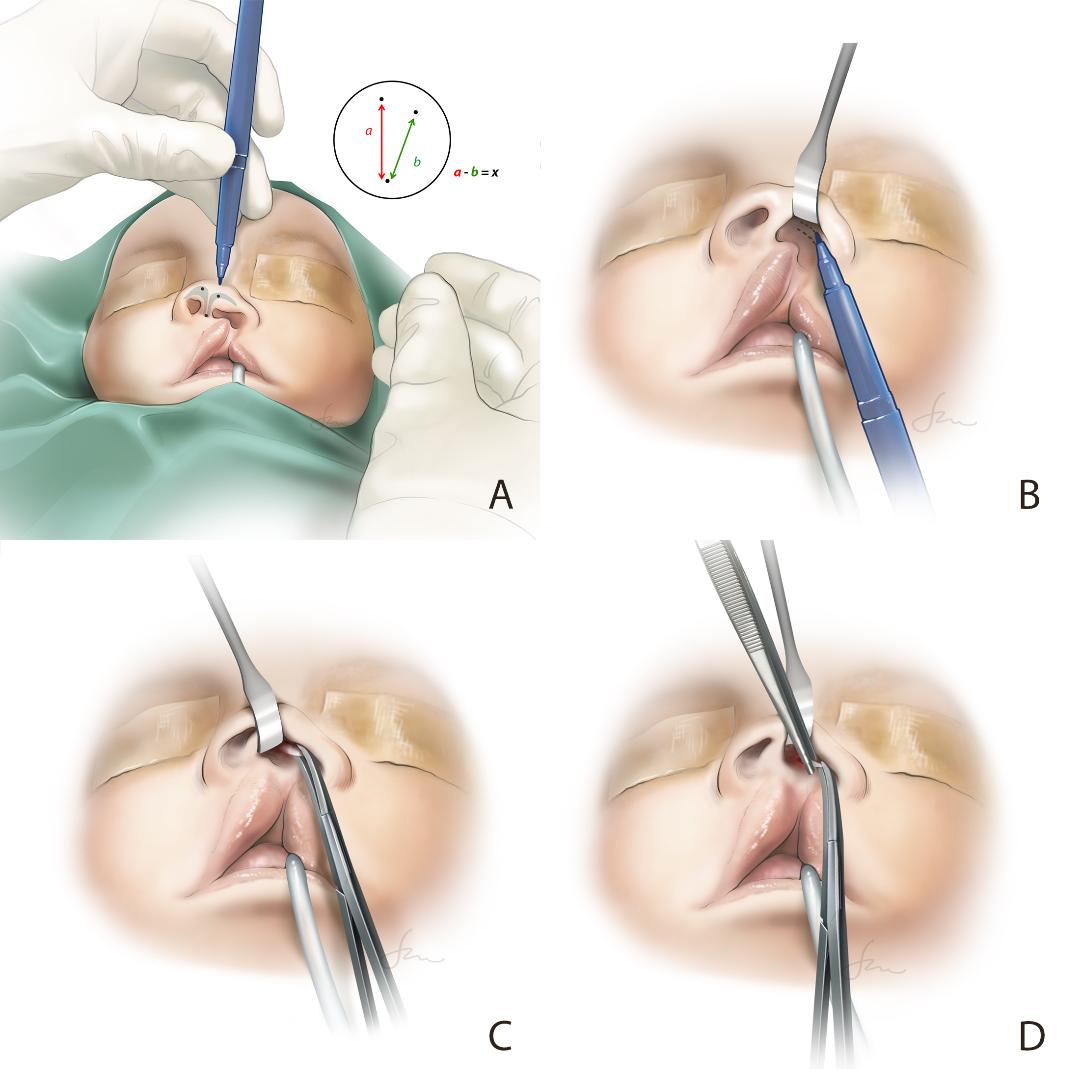


Supplementary material 3: Series of surgical steps showing the supero-medial repositioning of the cartilage composite complex and vermilion-vestibule graft inset


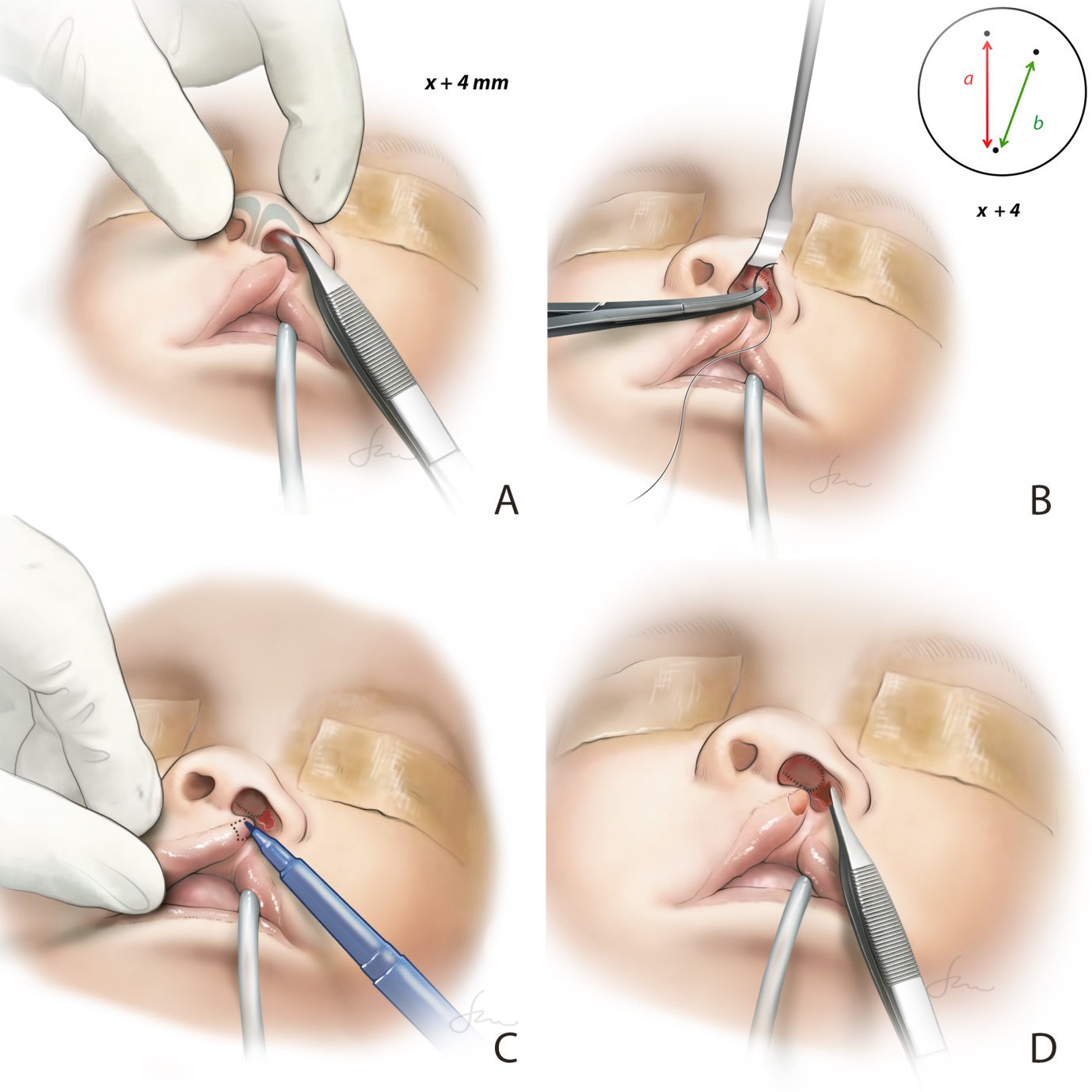


Supplementary material 4: Final reconstruction with immediate post op, and 1 year follow up

**
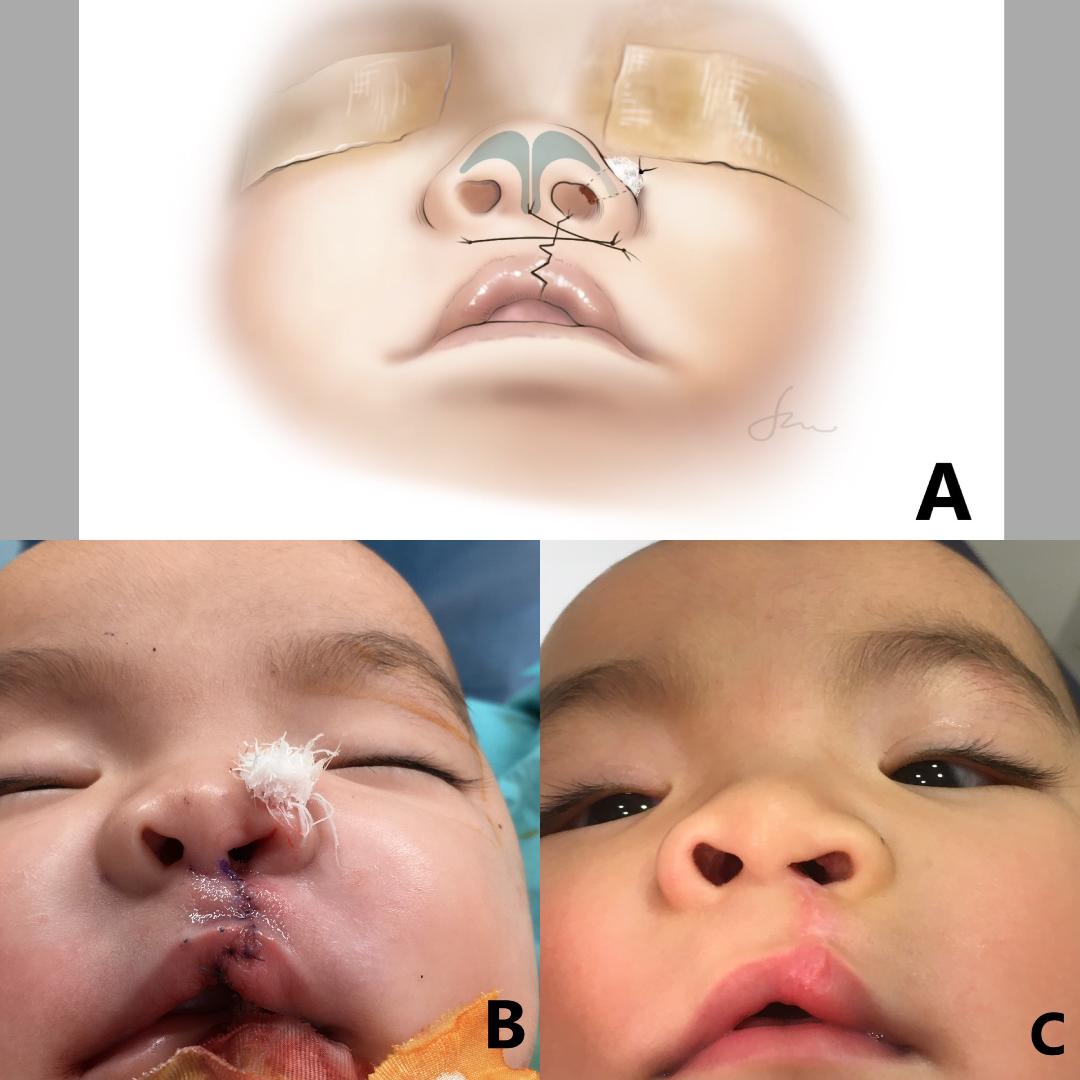
**
